# Multiple imputation methods for missing multilevel ordinal outcomes

**DOI:** 10.1186/s12874-023-01909-5

**Published:** 2023-05-09

**Authors:** Mei Dong, Aya Mitani

**Affiliations:** Division of Biostatistics, Dalla Lana School of Public Health, University of Toronto, Toronto, Canada

**Keywords:** Multiple imputation, Multilevel data, Ordinal outcomes, Generalized estimating equations, Informative cluster size

## Abstract

**Background:**

Multiple imputation (MI) is an established technique for handling missing data in observational studies. Joint modelling (JM) and fully conditional specification (FCS) are commonly used methods for imputing multilevel data. However, MI methods for multilevel ordinal outcome variables have not been well studied, especially when cluster size is informative on the outcome. The purpose of this study is to describe and compare different MI strategies for dealing with multilevel ordinal outcomes when informative cluster size (ICS) exists.

**Methods:**

We conducted comprehensive Monte Carlo simulation studies to compare the performance of five strategies: complete case analysis (CCA), FCS, FCS+CS (including cluster size (CS) in the imputation model), JM, and JM+CS under various scenarios. We evaluated their performance using a proportional odds logistic regression model estimated with cluster weighted generalized estimating equations (CWGEE).

**Results:**

The simulation results showed that including CS in the imputation model can significantly improve estimation accuracy when ICS exists. FCS provided more accurate and robust estimation than JM, followed by CCA for multilevel ordinal outcomes. We further applied these strategies to a real dental study to assess the association between metabolic syndrome and clinical attachment loss scores. The results based on FCS + CS indicated that the power of the analysis would increase after carrying out the appropriate MI strategy.

**Conclusions:**

MI is an effective tool to increase the accuracy and power of the downstream statistical analysis for missing ordinal outcomes. FCS slightly outperforms JM when imputing multilevel ordinal outcomes. When there is plausible ICS, we recommend including CS in the imputation phase.

**Supplementary Information:**

The online version contains supplementary material available at 10.1186/s12874-023-01909-5.

## Introduction

Multilevel ordinal outcomes commonly appear in observational studies. In a study of dental disease, maximum clinical attachment loss (CAL) – recorded on each tooth using an ordinal scoring system – is used to assess periodontal health. In this context, each subject contributes multiple outcomes of interest (CAL) and they are clustered within a subject. Therefore the correlation between the outcomes within the same subject needs to be accounted for in the analysis. Generalized linear mixed effect model (GLMM) and generalized estimating equations (GEE), which are both extensions of the generalized linear model (GLM), are the two most popular methods to model such multilevel data. GLMM gives cluster-specific inference while marginal models using GEE give population-average inference.

Both GLMM and GEE assume that cluster size (CS) and the outcome of interest are independent given the covariates. However, this assumption can be violated when CS changes with the degree of the outcome. For example, in periodontitis, the probability of losing a tooth increases with the severity of the disease. Hence patients with advanced periodontitis tend to have fewer number of teeth, or smaller CS, compared to patients with good oral health. This type of situation, when the outcome is dependent on CS conditional on the observed covariates, is referred to as informative cluster size (ICS). ICS also presents in many other research settings, such as in reproductive toxicology, neuroimaging data, etc. [1-4]. Examining the correlation coefficient between the outcome and cluster size is a common approach to detect ICS [5-7] as well as testing for the effect of cluster size in a model that regresses the outcome against cluster size and other predictors [5, 6]. To handle ICS, Seaman et al. summarized a number of methods based on GLMM and GEE [8]. Including the CS as a covariate in the model is one of the solutions, but it changes the interpretations of the other coefficients included in the model. When the interest is in making marginal inference, Williamson et al. and Benhin et al. both proposed cluster weighted GEE (CWGEE), which provides an unbiased estimator when ICS exists [9, 10] and can be used to model ordinal outcomes [11]. Based on CWGEE, Benhin et al. proposed a Wald test for ignorability of cluster sizes by comparing the estimators between GEE and CWGEE under the null hypothesis of igonorability [10].

While CAL cannot be measured on missing teeth (which leads to the issue of ICS), we observed that some CAL measurements were also missing on existing teeth for unknown reasons in the motivated dental study. Removing the teeth with missing CAL values from the analysis will produce inconsistency between CS and the number of observed outcomes for some of the subjects. Furthermore, marginal models using GEE assume that missing data are unrelated to observed and unobserved variables. Therefore when the interest is in making marginal inference, missing data problems are often dealt by multiple imputation (MI) [12, 13]. Briefly, MI involves three phases: imputation phase, analysis phase, and pooling phase. In the imputation phase, the missing values are filled with plausible values estimated from the posterior predictive model. This process is repeated *M* times, creating *M* different complete datasets. Then in the analysis phase, a statistical model is fitted to each of the *M* complete datasets, leading to *M* unique estimates and variance-covariance matrices of the parameters. Finally, in the pooling phase, the *M* parameter estimates and variances are pooled to create one set of parameters and variance estimates [14].

There are two main strategies for MI: joint modelling (JM) and fully conditional specification (FCS), also known as MI with chained equation (MICE). JM assumes that partially observed data follow a multivariate normal distribution [12]. Therefore, imputed categorical variables may have implausible values falling outside the range or in between the categories. Rounding off or latent normal variables was suggested to impute categorical data. However, rounding off continuous variable into categorical variable has been questioned for introducing bias in the estimates of interest [15]. FCS imputes each incomplete variable one at a time based on an imputation model that assumes the distribution of the variable. For example, missing values from a binary variable can be imputed from a logistic regression model. Hence FCS requires a unique specification of the imputation model for each variable with missing values [16]. Both strategies have been implemented in mainstream statistical programming languages including R and standalone software. R packages that perform MI based on JM include **norm** [17], **cat** [18], **mix** [19], **pan** [20] and **jomo** [21]. There is also a standalone software **REALCOM-IMPUTE** [22] that performs JM imputation. However, only **REALCOM-IMPUTE** and **jomo** handle multilevel categorical data through a latent normal approach. The R package **mice** is the most commonly used R package to implement FCS, which provides many options for model specification [23]. However, **mice** has limited options to impute multilevel data. Other packages, such as **micemd** [24] and **miceadds** [25], as extensions for **mice**, provide more options for different types of variables in multilevel data. Nevertheless, **micemd** does not deal with ordinal data and **miceadds** uses predictive mean matching to impute ordinal data. Recently, Enders et al. developed a standalone software **Blimp** that uses a latent probit approach to impute multilevel ordinal data [26], providing a better alternative to impute multilevel ordinal data using FCS.

To summarize, the R package **jomo** and the standalone software **Blimp** are two most popular tools to impute missing multilevel ordinal data. Although their performances on imputing multilevel continuous and binary data have been compared in many different aspects [27-29], their performances on imputing multilevel ordinal outcomes, especially when ICS exists, have not been studied yet. Kombo et al. compared FCS and JM for ordinal longitudinal data with monotone missing data patterns [30], but many multilevel data, such as clustered dental data, do not follow a monotone missing data pattern.

The objective of this paper was to compare the performances between JM and FCS when imputing multilevel ordinal outcomes that are subject to ICS. Two available software were used: **jomo** package in R for JM and **Blimp** for FCS. For each of the JM and FCS approaches, we additionally assessed whether the inclusion of CS in the imputation model improved parameter estimation of the analysis model. Since we were interested in the population-average inference, parameters in the analysis model were estimated by CWGEE with proportional odds logit. Extensive simulation studies were conducted to assess the performance of each imputation model under different scenarios and different missing patterns.

## Methods

### Motivating example: the VADLS study

Our study was motivated by the Veterans Affairs Dental Longitudinal Study (VADLS), which was a closed-panel longitudinal study that monitored oral health and diseases of male subjects from the greater Boston metropolitan area [31]. The health status of the subjects was measured approximately every three years. For illustration, we focused on one cycle of the longitudinal study, which included 241 subjects with a total of 5,100 teeth. We were interested in assessing the association between metabolic syndrome (MetS) and increasing CAL scores [32]. The baseline characteristics of the variables are given in Supplementary Table S1. CAL score was a level-1 (tooth/member level) ordinal variable of four categories (0: < 2mm, 1: 2-2.9mm,2: 3-4.9mm, 3: $\ge$ 5mm) with the higher score indicating worse prognosis of periodontal disease. We modelled the association between MetS (yes/no) and ordinal CAL scores using the proportional odds logistic regression model adjusting for the following level-2 (subject/cluster level) variables: age, smoking status (ever-smoker/never-smoker), and education levels (high school/some college/college graduate). These variables have been shown to be associated with CAL scores in previous studies [32, 33]. The marginal analysis model had the following form:

1 $$\begin{aligned} & \text{logit}\left\{\Pr(\text{CAL}_{ij} \leq c)\right\} = \eta_c + \beta_1 \text{MetS}_i +\beta_2 \text{age}_i \\ & + \beta_3 \text{smoking}_i + \beta_4 1(\text{edu}_i=\text{some college}) \\ &+\beta_5 1(\text{edu}_i=\text{college graduate}), \quad c=1,2,3 \end{aligned}$$

where $\text {CAL}_{ij}$ is the CAL score recorded on the *j*th tooth of the *i*th subject, $j=1,...,n_i$, $i=1,...,N$, $n_i$ is the CS (number of teeth) for subject *i*, and *N* is the total number of subjects. Two issues existed in producing valid inference from Equation (1). First, the number of teeth per subject ranged from 1 to 28. The Spearman correlation coefficient of the mean CAL score per subject and the number of teeth per subject was -0.41 (95% CI: (-0.44, -0.38)), indicating the presence of ICS in this data. Supplementary Fig. S1 shows that the mean CAL score decreased as the number of teeth per participant increased. Therefore, CWGEE was applied for estimation. Second, CAL scores were missing in 19% of all existing teeth. Hence, MI was applied to make use of all available data in model fitting. In addition to CAL, three other level-1 variables that measure the prognosis of periodontitis were available: probing pocket depth (PPD), radiographic alveolar bone loss (ABL), and tooth mobility (Mobil). PPD, ABL, and Mobil were also recorded using ordinal scoring systems and were correlated with each other as well as CAL. Although PPD, ABL, and Mobil were not included in the analysis model, they were included in the imputation model to help impute missing CAL.

### Ordinal regression with CWGEE

In the dental study, our goal was to obtain the marginal inference of the association between MetS and periodontal health of a typical tooth from a randomly selected subject. GEE is appealing not only because the estimator of $\varvec{\beta }$ is almost as efficient as the maximum likelihood estimator but also because it provides a consistent estimator of $\varvec{\beta }$ even under a misspecified within-subject association among the repeated measurements in sufficiently large samples [34]. Due to ICS, estimation using GEE will have oversampled healthy teeth, resulting in biased coefficient estimations [5]. To overcome this challenge, Williamson et al. and Benhin et al. proposed CWGEE [9, 10], which weighs the GEE by the inverse of CS. Let $Y_{ij}$ denote the ordinal outcome with $C>2$ categories and $\varvec{X}_{ij}=(X_{ij1},...,X_{ijp})^T$ denote the sets of *p* fixed covariates for the *j*th member (tooth) of the *i*th cluster (subject). The model for the ordinal outcome using proportional odds logistic regression is expressed as

$$\begin{aligned} \text {logit}\{\Pr (Y_{ij} \le c)\}=\eta _c+\varvec{X}_{ij}^T \varvec{\beta }, \quad c=1,...,C-1. \end{aligned}$$

For estimation, it is common to re-express the *C*-category outcome $Y_{ij}$ as a set of $C-1$ binary outcomes, such that

$$\begin{aligned} U_{ij,c} = \left\{ \begin{array}{ll} 1 &{} Y_{ij} \le c, \\ 0 &{} Y_{ij} > c \end{array}\right. \quad c=1,...,C-1 \end{aligned}$$

and write the model as a set of $C-1$ logistic regression models for each binary outcome [35]:

$$\begin{aligned} \text {logit}\{\text {E}(U_{ij,c})\}=\text {logit}\{\Pr (U_{ij,c} = 1)\}=\eta _c+\varvec{X}_{ij}^T \varvec{\beta }, \quad c=1,...,C-1. \end{aligned}$$

Let $\varvec{\mu _{ij}}=\text {E}(\varvec{U}_{ij})$, where $\varvec{U}_{ij} = (U_{ij,1}, ..., U_{ij,C-1})^{T}$. Then the estimations of $(\eta _1, ..., \eta _{C-1}, \varvec{\beta })$ are obtained by solving the following CWGEE,

2 $$\begin{aligned} \sum _{i=1}^N \frac{1}{n_i}\sum _{j=1}^{n_i}\varvec{D}_{ij}^{T}\varvec{V}_{ij}^{-1}(\varvec{U}_{ij}-\varvec{\mu }_{ij})=0, \end{aligned}$$

where $\varvec{D}_{ij}=\partial \varvec{\mu }_{ij}/{\partial \varvec{\beta }}$, $\varvec{V}_{ij}=\varvec{A}_{ij}^{1/2} \varvec{Q}_{ij} \varvec{A}_{ij}^{1/2}$ and $\varvec{A}_{ij}$ is the diagonal matrix containing the variance of $\varvec{U}_{ij}$, where $\text {Var}(\varvec{U}_{ij}) = \varvec{\mu }_{ij}(1-\varvec{\mu }_{ij})$, and $\varvec{Q}_{ij}$ includes the pairwise correlation between $U_{ij,c}$ and $U_{ij,c'}$ for $c, c'=1,...,C-1, c \ne c'$. Note that with these estimating equations, we assume a “working independence” structure between the teeth within a subject which is conventional in CWGEE [9]. A robust variance-covariance matrix $\varvec{\Psi }$ for $(\hat{\eta }_{1},...,\hat{\eta }_{C-1}, \hat{\varvec{\beta }})$ is estimated from the sandwich variance estimator, which has the form

$$\begin{aligned} \hat{\varvec{\Psi }}=\hat{\varvec{H}}^{-1} \hat{\varvec{M}} \hat{\varvec{H}}^{-1}, \end{aligned}$$

where

$$\begin{aligned} \hat{\varvec{H}}=\sum _{i=1}^N \frac{1}{n_i}\sum _{j=1}^{n_i}\hat{\varvec{D}}_{ij}^{T}\hat{\varvec{V}}_{ij}^{-1} \hat{\varvec{D}}_{ij}, \end{aligned}$$

and

$$\begin{aligned} \hat{\varvec{M}}=\sum _{i=1}^N \left\{ \frac{1}{n_i}\sum _{j=1}^{n_i}\hat{\varvec{D}}_{ij}^{T}\hat{\varvec{V}}_{ij}^{-1}(\varvec{U}_{ij}-\varvec{\hat{\mu }}_{ij})\right\} \left\{ \frac{1}{n_i}\sum _{j=1}^{n_i}\hat{\varvec{D}}_{ij}^{T}\hat{\varvec{V}}_{ij}^{-1}(\varvec{U}_{ij}-\varvec{\hat{\mu }}_{ij})\right\} ^{T}. \end{aligned}$$

### MI with multilevel ordinal data

MI is a Monte Carlo technique in which missing values are replaced by a set of simulated values drawn from the posterior predictive distribution $P(Y_{miss}|Y_{obs})$, where $Y_{miss}$ and $Y_{obs}$ are unobserved sample data and observed sample data, respectively. JM and FCS are two main strategies for MI: JM assumes a multivariate normal model for all variables while FCS specifies a unique model for each variable and imputes each of them sequentially. For either strategy, the imputation phase can be summarized into two steps for one single continuous incomplete variable [28]:

1. Draw $\varvec{\theta }$, the parameters of the imputation model, from the posterior distribution using complete data $P(\varvec{\theta }|Y_{obs})$;
2. Update the imputation by drawing plausible values for $Y_{miss}$ from the posterior predictive model $P(Y_{miss}|Y_{obs}, \varvec{\theta })$.

To sample parameters of the imputation model, Bayesian modelling is commonly employed by specifying a prior distribution of $\varvec{\theta }$. Gibbs sampler, an iterative computational algorithm, is often used to yield an empirical estimate of each parameter’s marginal posterior distribution [26]. For multilevel data, random effects are included in the imputation model to account for the correlation between members within the same cluster [26, 27].

Following the variables in the dental study, suppose we have a level-1 ordinal incomplete outcome variable $Y_{ij}$ (CAL) with $C_Y$ categories as well as three “auxiliary” level-1 ordinal variables, $M_{1,ij}$ (PPD), $M_{2,ij}$ (ABL), and $M_{3,ij}$ (Mobil) with $C_{M_1}$, $C_{M_2}$, and $C_{M_3}$ categories respectively. The auxiliary variables are not of direct interest in the analysis but can improve imputation accuracy if included in the imputation model. In addition, we have a level-2 continuous covariate $X_{i}$ and a level-2 binary covariate $Z_{i}$, which are both fully observed. These covariates are included in both the analysis and imputation models. In the next two subsections, we describe how data consisting of the aforementioned variables are multiply imputed with R package **jomo** and software **Blimp**.

#### Joint modelling with R package jomo

JM imputes missing data by assuming that partially observed variables follow a multivariate normal distribution. In the R package **jomo**, categorical variables are substituted with latent normal variables during Gibbs sampling and then converted back to discrete values using thresholds [21]. For categorical variables with *C* levels, we need $C-1$ latent normal variables, each of which has a fixed variance of 1 and covariance with the other latent normal variables of 0.5 [36]. To deal with multilevel data, a multivariate version of linear mixed effects model can be used. In this paper, we considered the random intercepts model since only the level-1 outcomes contained missing data. Let $Y_{ij,c}^*$, $M_{1,ij,c}^{*}, M_{2,ij,c}^{*}$, and $M_{3,ij,c}^{*}$ be the latent normal variables for $Y_{ij},M_{1,ij}, M_{2,ij}, M_{3,ij}$ in level *c* respectively. Then, we can construct a multivariate random intercepts model as the multilevel imputation model:

$$\begin{aligned} Y_{ij,c}^*&= \beta _{Y, 0}+\gamma _{Y, 1}X_i + \gamma _{Y, 2}Z_i+u_{Y,i,c}+\epsilon _{Y,ij,c}, c=1,...,C_Y-1 \\ M_{1,ij,c}^{*}&= \beta _{M_1,0}+\gamma _{M_1, 1}X_i + \gamma _{M_1,2}Z_i+u_{M_1,i,c}+\epsilon _{M_1,ij,c}, c=1,...,C_{M_1}-1 \\ M_{2,ij,c}^{*}&= \beta _{M_2,0}+\gamma _{M_2,1}X_i + \gamma _{M_2,2}Z_i+u_{M_2,i,c}+\epsilon _{M_2,ij,c}, c=1,...,C_{M_2}-1 \\ M_{3,ij,c}^{*}&= \beta _{M_3,0}+\gamma _{M_3,1}X_i + \gamma _{M_3,2}Z_i+u_{M_3,i,c}+\epsilon _{M_3,ij,c}, c=1,...,C_{M_3}-1 \end{aligned}$$

$$\begin{aligned} \text{with } & \left(\begin{array}{c} u_{Y,i, 1}\\ \dots \\ u_{Y,i, C_Y-1} \\ u_{M_1,i,1} \\ \dots \\ u_{M_3,i, C_{M_3}-1} \end{array}\right) \sim MVN(\boldsymbol{0}, \boldsymbol{\Sigma}_u), \\ \text{and } & \left(\begin{array}{c} \epsilon_{Y,ij,1}\\ \dots \\ \epsilon_{Y,ij,C_Y-1}\\ \epsilon_{M_1,ij,1} \\ \dots \\ \epsilon_{M_3,ij, C_{M_3}-1} \end{array}\right) \sim MVN(\boldsymbol{0}, \boldsymbol{\Sigma}_\epsilon) \end{aligned}$$

Then, the imputed latent variables are converted back to discrete values as follows:

$$\begin{aligned} Y_{ij}= \left\{ \begin{array}{ll} 1, &{} Y_{ij,1}^*> 0\ \& \ Y_{ij,1}^*> Y_{ij,c}^* \ \text {for} \ c \ne 1 \text { or } C_Y\\ 2, &{} Y_{ij,2}^*> 0\ \& \ Y_{ij,2}^* > Y_{ij,c}^* \ \text {for} \ c\ne 2 \text { or } C_Y \\ \dots \\ C, &{} Y_{ij,c}^* < 0\ \text {for}\ c \ne C_Y \end{array}\right. \end{aligned}$$

and similarly for $M_{1,ij}, M_{2,ij}, M_{3,ij}$.

#### Fully conditional specification with Blimp software

Instead of assuming variables with missing data follow a multivariate normal distribution, FCS assumes a unique distribution for each variable with missing values. Based on the distribution of the variable, a unique type of imputation model can be specified (e.g. linear regression for continuous variable, logistic regression for binary variable). The FCS procedure for our data can be summarized as follows for iteration *t*:

1. $Y_{ij}$ is drawn from the following distribution:
  $$\begin{aligned} Y_{ij(miss)}^{(t)} \sim N(&\beta _{Y,0}+\beta _{Y,1}M_{1,ij}^{(t-1)} +\beta _{Y,2}M_{2,ij}^{(t-1)} +\beta _{Y,3}M_{3,ij}^{(t-1)}+ \\&\gamma _{Y,1}X_{i}+\gamma _{Y,2}Z_{i}+u_{Y,0}, \sigma _{\epsilon ,Y}^2), \end{aligned}$$
  where $\sigma _{\epsilon ,Y}^2$ is the within-cluster residual variance.
2. After updating $Y_{ij}$, $M_{1,ij}$ is now treated as the outcome and $Y_{ij}$ and other covariates are treated as predictors. $M_{1,ij}$ is drawn from the following distribution:
  $$\begin{aligned} M_{1,ij(miss)}^{(t)} \sim N(&\beta _{M_1,0}+\beta _{M_1,1}Y_{ij}^{(t)} +\beta _{M_1,2}M_{2,ij}^{(t-1)} +\beta _{M_1,3}M_{3,ij}^{(t-1)}+\\&\gamma _{M_1,1}X_{i}+\gamma _{M_1,2}Z_{i}+u_{M_1,0}, \sigma _{\epsilon ,M_1}^2). \end{aligned}$$
3. After updating $M_{1,ij}$, repeat the above steps to update $M_{2,ij}$ and $M_{3,ij}$.

The above steps are standard for multilevel FCS, which are implemented in the R package **mice** [23]. This approach is flexible and useful in many applications, but the imputation model options in **mice** for incomplete level-1 variables are limited to continuous or binary variables. Enders et al. extended **mice** to handle incomplete nominal and ordinal variables and developed the software program **Blimp** [26].

In **Blimp**, for ordinal data, the cumulative probit model is used to link the distribution of latent variables to discrete outcomes using a threshold parameter. We can update the imputation model for $Y_{ij}$ in step 1 to:

3 $$\begin{aligned} Y_{ij(miss)}^{*(t)} \sim N(&\beta _{Y^*,0}+\beta _{Y^*,1}M_{1,ij}^{(t-1)} +\beta _{Y^*,2}M_{2,ij}^{(t-1)} +\beta _{Y^*,3}M_{3,ij}^{(t-1)}+\nonumber \\&\gamma _{Y^*,1}X_{i}+\gamma _{Y^*,2}Z_{i}+u_{Y^*,1}, 1). \end{aligned}$$

Then the imputed latent variable is converted to an ordinal variable using the following function:

$$\begin{aligned} Y_{ij(miss)}^{(t)}=f(Y_{ij(miss)}^{*(t)})= \left\{ \begin{array}{ll} 1, &{} -\infty<Y_{ij(miss)}^{*(t)}<\tau _1 \\ 2, &{} \tau _1<Y_{ij(miss)}^{*(t)}<\tau _2 \\ \dots \\ C, &{} \tau _{C-1}<Y_{ij(miss)}^{*(t)}<\infty , \end{array}\right. \end{aligned}$$

which finalize the imputation of *Y* in iteration *t*. We then repeat steps 2 and 3 similarly to other variables $M_{1,ij}$, $M_{2,ij}$, and $M_{3,ij}$ until all missing data are imputed.

#### Imputation model for data with ICS

In MI, the imputation model needs to include all the features of the analytical model [37]. The existence of ICS indicates that the outcome $Y_{ij}$ is dependent on $n_i$ and ignoring this relationship in the imputation model may lead to an inefficient and biased estimation of the posterior distribution. One way to deal with this is to include $n_i$ in the imputation model to account for the dependence between the missing $Y_{ij}$ values and $n_i$. Taken FCS as an example, $Y_{ij(miss)}^{*(t)}$ in Equation (3) can then be rewritten as

$$\begin{aligned} Y_{ij(miss)}^{*(t)} \sim N( &\beta_{Y^*,0}+\beta_{Y^*,1}M_{1,ij}^{(t-1)} +\beta_{Y^*,2}M_{2,ij}^{(t-1)} +\beta_{Y^*,3}M_{3,ij}^{(t-1)}+ \\ &\gamma_{Y^*,1}X_{i}+\gamma_{Y^*,2}Z_{i}+\gamma_{Y^*,3}n_{i}+u_{Y^*,1}, 1). \end{aligned}$$

Note that $n_i$ is only included in the imputation phase to account for additional variance of $Y_{ij}$, thus potentially improving the imputation accuracy. In the analysis model, ICS is accounted for by CWGEE as in Equation (2). In an extensive simulation study, we compared the effect of including versus omitting $n_i$ in the imputation model for both JM and FCS.

## Simulation studies

### Simulation setting

We evaluated the performance of five missing data approaches for multilevel ordinal outcomes with ICS through comprehensive Monte Carlo simulation studies: 1) Complete-case analysis (CCA); 2) FCS without CS as a predictor (FCS); 3) FCS with CS as a predictor (FCS+CS); 4) Joint modelling without CS as a predictor (JM); 5) Joint modelling with CS as a predictor (JM+CS).

To mimic the real dental data, we generated multilevel ordinal outcomes of $C=4$ categories similar to Equation (1). For the *j*th tooth of the *i*th subject, the outcome $Y_{ij}$ was simulated from the following equation:

4 $$\begin{aligned} &\text{logit}(\text{Pr}(Y_{ij} \le c))=\eta_c+\beta_1 X_i+\beta_2 Z_i, \\ & c=1,2,3, \quad j=1...,n_i, \quad i = 1,...,N, \end{aligned}$$

where $X_i$ was a level-2 continuous variable generated from $\text {N}(0, 2^2)$ and $Z_i$ was a level-2 binary variable generated from $\text {Binomial}(N, 0.5)$. The true values for the parameters were $(\eta _1, \eta _2, \eta _3,\beta _1, \beta _2 )=(-0.4, 0.8, 1.6, -0.2, -0.5)$. To make our simulation study more generalizable to other applications, we also simulated outcomes of $C=3$ categories with true values $(\eta _1, \eta _2, \beta _1, \beta _2 )=(-0.4, 0.8, -0.2, -0.5)$. In addition to outcome *Y*, we simulated three other auxiliary level-1 ordinal variables $M_{1}$, $M_{2}$, and $M_{3}$ following the same procedure with different values of intercepts $(\eta _1, \eta _2, \eta _3)$ and same value for $\beta _1$ and $\beta _2$ in Equation (4).

To simulate the ordinal outcome with ICS, we used the bridge distribution [11, 38] to obtain the marginal probability of success when fitting a proportional odds logistic regression model of the form:

$$\begin{aligned} p_{ij,c}&=\text{Pr}(U_{ij,c}=1|b_{ij}, X_{i},Z_{i}, \beta_1, \beta_2)\\ &=\frac{\exp \{ b_{ij} + (\eta_c+\beta_1 X_i+\beta_2 Z_i) \phi^{-1} \} }{1+\exp \{ b_{ij} + (\eta_c+\beta_1 X_i+\beta_2 Z_i) \phi^{-1} \}}, \end{aligned}$$

where $b_{ij}$ follows a bridge distribution with density $f_b(b_{ij} | \phi )=\frac{1}{2 \pi }\frac{\sin (\phi \pi )}{\cos (\phi b_{ij}) +\cos (\phi \pi )}$, $-\infty< b_{ij} < \infty$, $0< \phi <1$. The maximum CS of each subject was set to 28. We used the exchangeable correlation structure with parameter $\tau$ to simulate the correlation between teeth. For each subject *i*, we generated the baseline hazard $\lambda _i$ as a function of $\varvec{b}_i$, $\lambda _i=\frac{\exp (\nu \bar{b}_i))}{1+\exp (\nu \bar{b}_i))}$, where $\bar{b}_i=\sum _j \frac{b_{ij}}{n_i}$ with $\nu$ representing the degree of ICS. The number of teeth for each subject *i* was generated from $\text {Binomial}(28, \lambda _i)$. A detailed description of the simulation is shown in the Supplementary Materials.

We generated missing values through three different missing data mechanisms: missing completely at random (MCAR), missing at random (MAR), and missing not at random (MNAR) [39]. We further considered different levels of missingness on the outcome *Y*. To generate missing data, the missingness indicator $R_{ij}$ for the *j*th tooth of the *i*th subject were generated from the model:

$$\begin{aligned} \text {logit}\{\text {Pr}(R_{ij}=1)\}=\alpha _0+\alpha _1 X_{i} + \alpha _2 Z_{i} + \alpha _3 Y_{ij} + \alpha _4 M_{1,ij} +\alpha _5 M_{2,ij} + \alpha _6 M_{3,ij}. \end{aligned}$$

When $\alpha _1=\dots =\alpha _6=0$, data were MCAR. When only $\alpha _3=0$, data were MAR. Otherwise, data were MNAR. $\alpha _0$ was used to control the overall missing rate. For the outcome *Y*, we generated missing rates of 20% and 50%, representing low and high missing rates, respectively. For the auxiliary outcomes $M_{1}, M_{2}$, and $M_{3}$, the missing rates were 30%, 30%, 10%, respectively.

Table 1 shows the combination of the various parameters when data were MAR and $C=4$. We considered two different sample sizes *N*, 50 and 250. The degrees of correlation $\tau$ varied from 0, 0.1, 0.3, to 0.6, representing null, small, moderate, and strong correlations between teeth. We varied the degrees of ICS $\nu$ from 0, 0.1, to 0.4, representing null, moderate, and high correlation between the outcome and CS. When data were MCAR and MNAR, we considered the scenario where $N=50$ and 20% missing rate with varying ICC and degrees of ICS. Similarly, when the number of categories $C=3$, we simulated data with missing mechanism MAR with varying ICC and degrees of ICS. The missing rate was fixed at 20% and *N* was fixed at 50. We performed 1,000 replications for each scenario. We obtained the parameter estimates $(\hat{\eta }_1, \hat{\eta }_2, \hat{\eta }_3, \hat{\beta }_1, \hat{\beta }_2)$ from each simulated data in each scenario. The following metrics were computed to compare the performance of each imputation approach: (1) the mean of the parameter estimates (Mean Est); (2) the mean of the robust standard error estimates (Mean SEs); (3) the standard deviation of the parameter estimates (Empirical SEs); (4) the mean relative bias $\times$ 100% (Rel Bias); (5) the 95% coverage probability (Cov Prob); (6) the mean squared error (MSE).

**Table 1:** Parameter settings in the simulation study when data were MAR and $C=4$

Parameter	Notation	Values			
Sample size	N	50	250		
Degree of correlation (ICC)	$\tau$	0	0.1	0.3	0.6
Degree of ICS	$\nu$	0	0.1	0.4	
Missing rate	t	20%	50%		

Simulations for JM were performed with R software **jomo** and simulations for FCS were performed with software **Blimp**. For each replication, we created 5 imputed dataset. We set the burn-in iterations to be 4,000 and the iterations between two successive imputations to be 1,000 for both approaches.

### Results

**Fig. 1:**
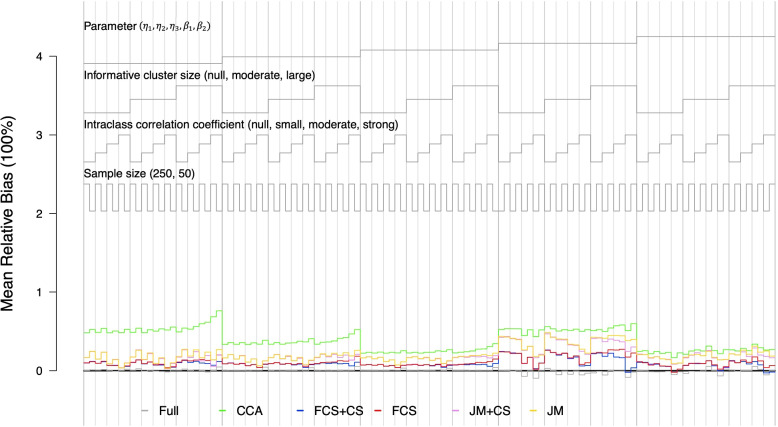
Mean relative bias of each imputation method and each parameter under different simulation scenarios. The missing data mechanism was MAR and $C=4$. The missing rate was 20%. Each column represents one combination of parameters of interest, degrees of ICS, and ICC, with two different sample sizes. The black line is the reference line at 0; the grey line represents the results using the full data; the green line represents the results using complete case analysis; the blue line represents the results using FCS+CS; the red line represents the results using FCS; the purple line represents the results using JM+CS; the orange line represents the results using JM

The nested loop plot in Fig. 1 shows the mean relative biases for $(\hat{\eta }_1, \hat{\eta }_2, \hat{\eta }_3, \hat{\beta }_1, \hat{\beta }_2)$ under various combinations of the simulation parameters listed in Table 1 when the missing mechanism was MAR, $C=4$, and missing rate was 20%. Each column represents two ($N=50$ and $N=250$) of the 120 combinations of simulation setting (5 parameters of interest $\times$ 3 degrees of ICS $\times$ 4 ICC $\times$ 2 sample sizes) and each color represents one of the five imputation approaches. The gray line represents the results from the full data with no missing values. The mean relative biases estimated from the CWGEE with full data were close to 0. Overall, CCA had the largest relative bias, followed by JM and JM+CS across all parameters. FCS+CS had the smallest mean relative bias in most scenarios, followed by FCS. By fixing the parameters of interest, ICC, and sample size, FCS and FCS+CS, JM and JM+CS almost overlapped when the degree of ICS was null or moderate. When the degree of ICS was large, both FCS+CS and JM+CS had smaller mean relative biases than FCS and JM. This indicated that including CS as a covariate in the imputation model for both FCS and JM improved the estimation accuracy when the degree of ICS was large, while including CS in the imputation phase when the degree of ICS was either null or moderate would not have a negative effect on the estimation accuracy. Fixing the parameters of interest, the degree of ICS, and sample size, the mean relative biases from FCS+CS, FCS, JM+CS, and JM decreased as ICC increased when the degree of ICS was null or moderate. However, when the degree of ICS was large, the relative biases did not change significantly for each MI approach regardless of the change in ICC. This suggested that the strong degree of ICS dominated the imputation accuracy rather than ICC. By looking at each column in Fig. 1, the mean relative bias slightly decreased when the sample size decreased from 250 to 50. The overall missing rate had a large impact on mean relative biases. The mean relative biases for scenarios where the missing rate was 50% were around two times larger than those under 20% missing rate (Supplementary Fig. S2). The difference was more considerable for JM+CS, JM, and CCA, suggesting that FCS was more reliable than JM when the missing rate was high. The missing rate in each level was approximately equal under each scenario. When the overall missing rate was 20%, the missing rate in each level across all simulation scenarios over 1,000 replications had an average of 7% (sd=0.007) in category 1, 17% (sd=0.015) in category 2, 27% (sd=0.019) in category 3, and 45% (sd=0.015) in category 4. When the overall missing rate was 50%, the missing rate in each level across all simulation scenarios over 1,000 replications had an average of 30% (sd=0.018) in category 1, 50% (sd=0.021) in category 2, 64% (sd=0.017) in category 3, and 80% (sd=0.005) in category 4. The small sd’s showed that the missing rate in each category over different replications and different simulation settings was primarily affected by the overall missing rate rather than other simulation settings.

Simulation results of intercept $\eta _1$ and slope $\beta _1$ for the scenario when the degree of ICS=0.1, ICC=0.3, missing rate was 20%, N=50, $C=4$, and missing mechanism was MAR are shown in Table 2. The mean estimate of FCS+CS or FCS for the intercept $\eta _1$ was -0.37, which was closer to the true value compared to JM+CS or JM. The mean relative biases were 7.06% and 7.98% for FCS and FCS+CS, respectively. The mean relative biases for slope $\beta _1$ were larger for all methods, including the estimate from full data. The mean SE and empirical SE for all approaches were close to 0.20. The coverage probabilities of FCS+CS, FCS, JM+CS, JM were all close to 95%. The MSE for CCA was 0.09, which was the largest for the intercept $\eta _1$, followed by JM+CS and JM. The difference between MSE for slope $\beta _1$ was smaller, while FCS+CS and FCS had similar MSE’s as the full data. The small difference in evaluation metrics between FCS+CS and FCS, JM+CS and JM indicated that including CS in the imputation did not significantly improve the estimation accuracy when the degree of ICS was moderate. Under the more extreme situation where the degree of ICS was large (the degree of ICS = 0.4) and ICC was strong (ICC=0.6) (Supplementary Table S2), we observed that including CS as a covariate in the imputation model shifted the mean $\hat{\eta }_1$ closer to the true value by 0.03 compared to omitting CS in the imputation model for both FCS and JM. Although the mean SE, empirical SE, and MSE in Supplementary Table S2 all increased compared to Table 2, they were still comparable to the full data except for CCA. MI approaches still drastically decreased the bias and increased the power under large degree of ICS and strong ICC.

**Table 2:** Results of intercept $\eta _1$ and slope $\beta _1$ when the degree of ICS=0.1, ICC=0.3, missing rate was 20%, sample size *N* was 50, missing mechanism was MAR, $C=4$

Parameter	Method	Mean Est	Mean SE	Empirical SE	Rel Bias (%)	Cov Prob (%)	MSE
$$\varvec{\eta _1=-0.4}$$							
	Full	-0.40	0.20	0.20	-0.97	95.50	0.04
	CCA	-0.19	0.21	0.22	53.07	79.70	0.09
	FCS+CS	-0.37	0.21	0.20	7.06	95.80	0.04
	FCS	-0.37	0.21	0.20	7.98	95.90	0.04
	JM+CS	-0.34	0.23	0.22	15.09	94.18	0.05
	JM	-0.33	0.23	0.22	16.31	95.08	0.05
$$\varvec{\beta _1=-0.2}$$							
	Full	-0.21	0.19	0.20	-4.48	92.40	0.04
	CCA	-0.10	0.21	0.23	51.09	89.90	0.06
	FCS+CS	-0.17	0.21	0.21	15.09	95.10	0.04
	FCS	-0.17	0.21	0.21	16.22	94.70	0.04
	JM+CS	-0.13	0.23	0.24	32.69	93.99	0.06
	JM	-0.13	0.24	0.24	33.50	93.57	0.06

Under the same simulation setting when data were MAR, we evaluated the performance of MI approaches when the number of categories was $C=3$ with different degrees of ICS and ICC. As shown in Supplementary Fig. S3, the mean relative biases were slightly smaller than the case when $C=4$, indicating that the imputation accuracy decreased as the number of outcome categories increased. We also considered the model misspecification in the imputation phase when $C=4$. We excluded the auxiliary variables that were used to simulate missing indicators for MAR in the imputation phase. As shown in Supplementary Fig. S4, the mean relative biases for all MI approaches increased compared to the case of having correct imputation model. In some cases when both the degree of ICS and ICC were null, the relative biases of MI approaches were close to CCA. This suggested that all approaches would be affected by the misspecification of the imputation model. However, FCS still outperformed JM when there was model misspecification in the imputation phase.

**Fig. 2:**
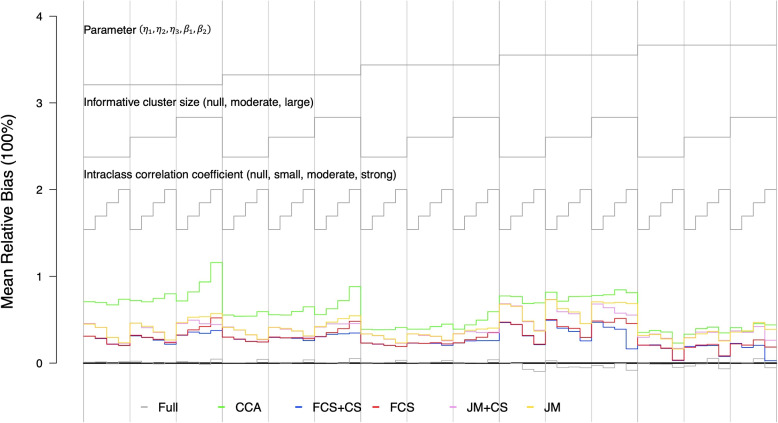
Mean relative bias of each imputation method and each parameter under different simulation scenarios. The missing data mechanism was MNAR and $C=4$. The missing rate was 20% and the sample size was 50. Each column represents one combination of parameters of interest and degree of ICS, with four different values of ICC. The black line is the reference line at 0; the grey line represents the results using the full data; the green line represents the results using complete case analysis; the blue line represents the results using FCS+CS; the red line represents the results using FCS; the purple line represents the results using JM+CS; the orange line represents the results using JM

We further investigated the impact of ICC and the degree of ICS on different MI approaches when data were MNAR and MCAR. Figure 2 shows the mean relative bias when the missing data mechanism was MNAR. The performance of FCS and JM also had an analogous pattern when ICC and the degree of ICS changed compared to when data were MAR. As shown in Table 3, the relative biases from FCS+CS and FCS were 26.5% and 27.5% respectively, with coverage probabilities close to 93%. The performance of FCS was still better than JM and CCA. When the degree of ICS and ICC changed, the change in relative bias was similar to our observation in Fig. 1. When the missing data mechanism was MCAR, all imputation methods yielded unbiased estimates for intercepts and slightly biased estimates for the slopes (Supplementary Fig. S5, Supplementary Table S3). Under the most extreme case, the biases of all MI approaches were approximately equally small (Supplementary Fig. S5).

**Table 3:** Results of intercept $\eta _1$ and slope $\beta _1$ when the degree of ICS=0.1, ICC=0.3, missing rate was 20%, sample size *N* was 50, missing mechanism was MNAR, $C=4$

Parameter	Method	Mean Est	Mean SE	Empirical SE	Rel Bias (%)	Cov Prob (%)	MSE
$$\varvec{\eta _{1}=-0.4}$$							
	Full	-0.40	0.20	0.20	-0.97	95.50	0.04
	CCA	-0.10	0.22	0.24	74.61	71.21	0.15
	FCS+CS	-0.29	0.23	0.22	26.52	93.38	0.06
	FCS	-0.29	0.23	0.22	27.51	92.89	0.06
	JM+CS	-0.26	0.25	0.25	35.41	91.44	0.08
	JM	-0.26	0.25	0.25	36.02	91.40	0.09
$$\varvec{\beta _{1}=-0.2}$$							
	Full	-0.21	0.19	0.20	-4.48	92.40	0.04
	CCA	-0.05	0.24	0.26	76.77	87.86	0.09
	FCS+CS	-0.13	0.24	0.22	36.58	96.09	0.05
	FCS	-0.12	0.24	0.22	40.06	95.40	0.05
	JM+CS	-0.09	0.28	0.25	56.94	94.47	0.08
	JM	-0.08	0.28	0.26	59.03	94.03	0.08

## Real data application

We used data from one cycle of the VADLS to compare the five missing data approaches. Based on the analysis model in Equation (1), we incorporated the level-1 variables described above, in addition to PPD, ABL, and Mobil. PPD, ABL, and Mobil are commonly used to quantify the severity of periodontitis, and are correlated with each other. Hence, they were included as auxiliary variables to improve the imputation accuracy for CAL. In the imputation phase, we implemented FCS+CS, FCS, JM+CS, JM, and CCA.

Table 4 summarizes the results from the VADLS data. We focus on the estimates of MetS. The ICC was 0.10 (95% CI: (-0.06, 0.17)) calculated using the two-way ANOVA fixed effect model [40], indicating small ICC in the VADLS data. The Spearman correlation being -0.41 indicates that there exists ICS, hence cluster size should be included in the imputation phase. Based on the simulation results, we focus on the results from FCS+CS. FCS+CS provided the narrowest confidence interval. The odds ratio of MetS is 0.82 (95% CI: (0.55, 1.22)), indicating that the odds of having a lower/healthier CAL score was approximately 20% lower for patients without MetS compared to patients with MetS. Recent studies have also reported the association between MetS and periodontal disease [41]. However, such an association would not have been found if CCA had been used in our data (OR = 1.01, 95% CI: (0.66, 1.55)). Other MI approaches (FCS, JM+CS, and JM) all tend to overestimate the effect size of MetS on CAL score.

**Table 4:** The odds ratio (95% CI) estimations for variables in the VADLS data based on different MI approaches

Method	Age	Smoking	Edu (some college)	Edu (college graduate)	MetS
CCA	0.96 (0.93, 0.99)	0.77 (0.46, 1.28)	0.96 (0.57, 1.61)	1.50 (0.86, 2.59)	1.01 (0.66, 1.55)
FCS+CS	0.96 (0.93, 0.99)	0.72 (0.41, 1.27)	1.02 (0.64, 1.62)	1.59 (0.97, 2.64)	0.82 (0.55, 1.22)
FCS	0.96 (0.93, 0.98)	0.65 (0.39, 1.09)	0.94 (0.59, 1.51)	1.49 (0.90, 2.47)	0.87 (0.57, 1.33)
JM+CS	0.96 (0.93, 0.99)	0.74 (0.43, 1.28)	1.07 (0.66, 1.73)	1.67 (1.00, 2.78)	0.89 (0.59, 1.34)
JM	0.96 (0.94, 0.99)	0.77 (0.46, 1.29)	0.96 (0.60, 1.55)	1.53 (0.92, 2.54)	0.83 (0.55, 1.24)

## Conclusions

In this study, we compared FCS, JM and CCA for imputing missing multilevel ordinal outcomes under different scenarios. Comprehensive simulation studies showed that FCS performed better than JM and CCA. FCS provided more reliable and stable performances with varying degrees of ICS, ICC, and missing rates. Including CS as a covariate in the imputation model improved the estimation accuracy when the degree of ICS was large. When there was no ICS, including CS in the imputation phase did not affect the results negatively. MI methods were valid only when the missing data mechanism was MCAR or MAR. Nevertheless, both JM and FCS performed better than CCA even when the missing data mechanism was MNAR.

Despite the comprehensive comparison between FCS and JM under different degrees of ICS, ICC, sample size, missing rate, and missing mechanism in our simulation study, choosing an optimal imputation model for multilevel ordinal outcomes is still complicated. Based on our simulation results, some strategies could be taken to minimize bias. First, CS should be included in the imputation model in the presence of ICS. Second, the sample size of the data set should increase as the missing rate and the number of categories of ordinal outcome increase to ensure the accuracy of the imputation model. Third, selecting the right imputation model is crucial to achieve high imputation accuracy. When there is model misspecification in the imputation phase, all MI approaches will produce biased results. Model misspecification is a special case of MNAR, where the predictors associated with missingness are not included in the imputation model. In practice, we recommend performing a sensitivity analysis if additional auxiliary variables are available. Expertise/clinical knowledge would also be important in choosing the optimal imputation model.

Although both FCS and JM are based on Monte Carlo techniques, they are fundamentally different. On the one hand, FCS implemented by **Blimp** imputes ordinal data using a threshold-based latent probit approach. Since it imputes one variable at a time, it is more flexible and easier to adjust to different data types. On the other hand, the R package **jomo** uses a nominal probit model, even for imputing ordinal data. Simulation studies conducted by Quartagno et al. showed that if the variable is truly ordinal, it gives good results with only a marginal loss in efficiency [36]. However, we observed that the bias from **jomo** was not negligible compared to FCS when ICS existed. There is another software **REALCOM-IMPUTE** that implements JM that could deal with incomplete multilevel ordinal outcomes, but unfortunately, it is only available for Windows users [22] and was not considered in this paper. In addition to the imputation accuracy, **Blimp** is also computationally faster than **jomo**. Creating 5 imputed dataset with 4,000 burn-in iterations and 1,000 iterations between two successive imputations costs the R package **jomo** 25 minutes, while only 21 seconds for software **Blimp** for imputing the real dental data with 241 samples using an M1 Mac with 8G RAM. However, both approaches are computationally affordable when imputing one dataset.

Our study has several limitations. First, we only considered the level-1 outcomes to be missing in our data. Missing level-1 and level-2 data could make the imputation more complicated. Comparing the performances of JM and FCS when both covariates and outcomes are missing under ICS could be one of the future works. Second, although this study found that the bias introduced by JM using the R package **jomo** was not negligible, it is not fair to conclude that JM is worse than FCS when imputing multilevel ordinal outcomes. Extending the package to deal with ordinal data could be considered. Third, we only considered the effect of subject-level (level-2) variables on the outcome. In many cases, tooth-level (level-1) predictors and the interaction effects between tooth-level and subject-level predictors are of interest. However, CWGEE may not perform well when including tooth-level predictors with the presence of ICS [42]. Hence, it imposes challenges to make reasonable comparisons of MI approaches when ICS exists. Future work can include tooth-level predictors and the interaction effects by applying an appropriate analysis model. Last but not least, our study focused on two-level clustered data and did not assess the time effect typically observed in longitudinal studies. Wijesuriya et al. compared the methods for three-level data with time-varying CS for continuous outcomes and exposures [29]. It would be of interest to extend the comparison to the case when ordinal outcomes are informative on cluster size.

To conclude, our study compared JM and FCS for imputing multilevel ordinal outcomes when data is subject to ICS. We found that FCS is currently the optimal choice, and we recommend including CS in the imputation model if there is potential for ICS. Our study provides further guidelines on choosing the imputation method when imputing multilevel ordinal outcomes.

## Supplementary Information

**Additional file 1.**

## Data Availability

The simulation program in the form of R code used to support the findings of this study is available in the MI-Multilevel-Ordinal repository, https://github.com/MeiDongstat/MI-Multilevel-Ordinal. The portion of the data that was used for illustration is available from the corresponding author upon reasonable request. The full data are available from the Dental Longitudinal Study and Normative Aging Study, which are components of the Massachusetts Veterans Epidemiology Research and Information Center, supported by the US Department of Veterans Affairs Cooperative Studies Program.

## Abbreviations

CAL: Clinical attachment loss
GLMM: Generalized linear mixed effects model
GEE: Generalized estimating equations
CS: Cluster size
ICS: Informative cluster size
CWGEE: Cluster weighted GEE
MI: Multiple imputation
JM: Joint modelling
FCS: Fully conditional specification
MAR: Missing at random
MCAR: Missing completely at random
MNAR: Missing not at random
VADLS: Veterans Affairs Dental Longitudinal Stud
PPD: Probing pocket depth
ABL: Radiographic alveolar bone loss
Mobil: Tooth mobility

## References

[1] Hoffman EB, Sen PK, Weinberg CR (2001). Within-cluster resampling. Biometrika..

[2] Dutta S (2022). Robust Testing of Paired Outcomes Incorporating Covariate Effects in Clustered Data with Informative Cluster Size. Stats..

[3] Shen B, Chen C, Chinchilli VM, Ghahramani N, Zhang L, Wang M (2022). Semiparametric marginal methods for clustered data adjusting for informative cluster size with nonignorable zeros. Biom J..

[4] Williamson JM, Kim HY, Warner L (2007). Weighting condom use data to account for nonignorable cluster size. Ann Epidemiol..

[5] Seaman S, Pavlou M, Copas A (2014). Review of methods for handling confounding by cluster and informative cluster size in clustered data. Stat Med..

[6] Pavlou M, Ambler G, Omar RZ (2021). Risk prediction in multicentre studies when there is confounding by cluster or informative cluster size. BMC Med Res Methodol..

[7] Mitani AA, Kaye EK, Nelson KP (2022). Accounting for drop-out using inverse probability censoring weights in longitudinal clustered data with informative cluster size. Ann Appl Stat..

[8] Seaman SR, Pavlou M, Copas AJ (2014). Methods for observed-cluster inference when cluster size is informative: a review and clarifications. Biometrics..

[9] Williamson JM, Datta S, Satten GA (2003). Marginal analyses of clustered data when cluster size is informative. Biometrics..

[10] Benhin E, Rao JNK, Scott AJ (2005). Mean estimating equation approach to analysing cluster-correlated data with nonignorable cluster sizes. Biometrika..

[11] Mitani AA, Kaye EK, Nelson KP (2019). Marginal analysis of ordinal clustered longitudinal data with informative cluster size. Biometrics..

[12] Schafer JL. Analysis of incomplete multivariate data. London: Chapman & Hall/CRC; 1997.

[13] Little RJ, Rubin DB. Statistical analysis with missing data. 2nd ed. New York: John Wiley & Sons; 2002.

[14] Rubin DB. Multiple imputation for nonresponse in surveys. New York: John Wiley & Sons; 2004.

[15] Horton NJ, Lipsitz SR, Parzen M (2003). A potential for bias when rounding in multiple imputation. Am Stat..

[16] van Buuren S. Flexible Imputation of Missing Data. 2nd ed. London: Chapman and Hall/CRC; 2018.

[17] Novo A. Schafer J. norm: Analysis of Multivariate Normal Datasets with Missing Values. R package version 1.0-10.0. 2022.

[18] Harding T, Tusell F, Schafer J. cat: Analysis of categorical-variable datasets with missing values. R package version 0.0-7. 2012.

[19] Schafer J. mix: Estimation/multiple Imputation for Mixed Categorical and Continuous Data. R package version 1.0-11. 2010.

[20] Zhao J, Schafer J. pan: Multiple imputation for multivariate panel or clustered data. R package version 1.6; 2018.

[21] Quartagno M, Grund S, Carpenter J. Jomo: a flexible package for two-level joint modelling multiple imputation. R J. 2019;11(2):205–28.

[22] Carpenter JR, Goldstein H, Kenward MG (2011). REALCOM-IMPUTE software for multilevel multiple imputation with mixed response types. J Stat Softw..

[23] Van Buuren S, Groothuis-Oudshoorn K. Mice: Multivariate imputation by chained equations in R. J Stat Softw. 2011;45(3):1–67.

[24] Audigier V, Resche-Rigon M. Micemd: Multiple imputation by chained equations with multilevel data. R package; 2017. 10.1177/0962280216666564 PMC5496677 27647809

[25] Robitzsch A, Grund S, Henke T. Miceadds: some additional multiple imputation functions, especially for ‘mice’. R package version 1.7–8. 2016.

[26] Enders CK, Keller BT, Levy R (2018). A fully conditional specification approach to multilevel imputation of categorical and continuous variables. Psychol Methods..

[27] Enders CK, Mistler SA, Keller BT (2016). Multilevel multiple imputation: A review and evaluation of joint modeling and chained equations imputation. Psychol Methods..

[28] Audigier V, White IR, Jolani S, Debray TP, Quartagno M, Carpenter J (2018). Multiple imputation for multilevel data with continuous and binary variables. Stat Sci..

[29] Wijesuriya R, Moreno-Betancur M, Carlin J, De Silva AP, Lee KJ. Multiple imputation approaches for handling incomplete three-level data with time-varying cluster-memberships. Stat Med. 2022;41(22):4385-402. 10.1002/sim.9515 PMC9540355 35893317

[30] Kombo AY, Mwambi H, Molenberghs G (2017). Multiple imputation for ordinal longitudinal data with monotone missing data patterns. J Appl Stat..

[31] Kapur KK, Glass RL, Loftus ER, Alman JE, Feller RP (1972). The Veterans Administration longitudinal study of oral health and disease: methodology and preliminary findings. Aging Hum Dev..

[32] Kaye E, Chen N, Cabral H, Vokonas P, Garcia R (2016). Metabolic syndrome and periodontal disease progression in men. J Dent Res..

[33] Gamonal J, Mendoza C, Espinoza I, Munoz A, Urzua I, Aranda W (2010). Clinical attachment loss in Chilean adult population: first Chilean national dental examination survey. J Periodontol..

[34] Fitzmaurice G, Davidian M, Verbeke G, Molenberghs G. Longitudinal data analysis. London: Chapman & Hall/CRC; 2008.

[35] Kenward MG, Lesaffre E, Molenberghs G (1994). An Application of Maximum Likelihood and Generalized Estimating Equations to the Analysis of Ordinal Data from a Longitudinal Study with Cases Missing at Random. Biometrics..

[36] Quartagno M, Carpenter JR (2019). Multiple imputation for discrete data: Evaluation of the joint latent normal model. Biom J..

[37] Sterne JA, White IR, Carlin JB, Spratt M, Royston P, Kenward MG, et al. Multiple imputation for missing data in epidemiological and clinical research: potential and pitfalls. BMJ. 2009;338:157–60. 10.1136/bmj.b2393 PMC2714692 19564179

[38] Parzen M, Ghosh S, Lipsitz S, Sinha D, Fitzmaurice GM, Mallick BK (2011). A generalized linear mixed model for longitudinal binary data with a marginal logit link function. Ann Appl Stat..

[39] Rubin DB (1976). Inference and missing data. Biometrika..

[40] Liljequist D, Elfving B, Skavberg Roaldsen K (2019). Intraclass correlation-A discussion and demonstration of basic features. PLoS ONE..

[41] Lamster IB, Pagan M (2017). Periodontal disease and the metabolic syndrome. Int Dental J..

[42] Huang Y, Leroux B (2011). Informative cluster sizes for subcluster-level covariates and weighted generalized estimating equations. Biometrics..

