# Supplementary material for: Multiple imputation methods for missing multilevel ordinal outcomes
Source: BMC Med Res Methodol. 2023 May 9;23:112. doi: 10.1186/s12874-023-01909-5 (PMC10169455; doi:10.1186/s12874-023-01909-5)

Supplementary Materials for "Multiple Imputation Methods for Missing Multilevel Ordinal Outcomes"

Mei Dong, Aya Mitani

1 Details on Simulating Multilevel Ordinal Data with Informative Cluster Size

For every cluster i,

1. Sample $\boldsymbol{\omega}_i = (\omega_{i1}, \ldots, \omega_{im})'$ from a multivariate normal distribution, with mean vector $\mathbf{0}$ and variance matrix $\boldsymbol{\Sigma}$, where

$$\Sigma = \begin{pmatrix} 1 & \tau & \ldots & \tau \\ \tau & 1 & \ldots & \tau \\ \vdots & \vdots & \ddots & \vdots \\ \tau & \ldots & \tau & 1 \end{pmatrix}_{mxm}$$

   τ is the correlation between each pair of units within a cluster. We used the exchangeable correlation structure to generate correlation between teeth.

2. Compute $\boldsymbol{u}_i = \Phi(\boldsymbol{\omega}_i)$, where Φ is the CDF of the standard normal distribution.

3. Compute $\boldsymbol{b}_i = \frac{1}{\phi} \log \frac{sin(\phi\pi\boldsymbol{u}_i)}{sin(\phi\pi(1-\boldsymbol{u}_i))}$, with $\phi = 0.5$. b_{ij} has marginal bridge distribution and b_{ij} and b_{ik} are correlated due to the correlation imposed by ω_{ij}.

4. Compute the baseline level of risk λ_i for each cluster such that $\lambda_i = \frac{\exp(\nu\bar{b}_i)}{1+\exp(\nu\bar{b}_i))}$, where $\bar{b}_i = \sum_j \frac{b_{ij}}{n_i}$.

5. Sample CS n_i from a truncated Bin$(28, \lambda_i)$.

6. Generate the outcome Y_{ij}, which takes values from 1, 2, 3, and 4 from a multinomial distribution with a set of probability $(P_{ij,1}, P_{ij,2}, P_{ij,3}, P_{ij,4})$ such that

$$P_{ij,1} = \Pr(Y_{ij} = 1 | b_{ij}, X_i, Z_i, \beta_1, \beta_2) = \theta_1$$

$$P_{ij,2} = \Pr(Y_{ij} = 2 | b_{ij}, X_i, Z_i, \beta_1, \beta_2) = \theta_2 - \theta_1$$

$$P_{ij,3} = \Pr(Y_{ij} = 3 | b_{ij}, X_i, Z_i, \beta_1, \beta_2) = \theta_3 - \theta_2$$

$$P_{ij,4} = \Pr(Y_{ij} = 4 | b_{ij}, X_i, Z_i, \beta_1, \beta_2) = 1 - \theta_3,$$

where $\theta_c = \frac{\exp\{b_{ij}+(\eta_c+\beta_1 X_i+\beta_2 Z_i)\phi^{-1}\}}{1+\exp\{b_{ij}+(\eta_c+\beta_1 X_i+\beta_2 Z_i)\phi^{-1}\}}$, $c = 1, 2, 3$.

7. Repeat for $i = 1, \ldots, N$ subjects.

8. Repeat the whole process for each auxiliary outcome with different values of η_c.

2 Supplementary Tables

Table S1: Baseline Characteristics of variables. PPD, ABL and Mobil are auxiliary variables used in the imputation phase.

Variables	Type	Categories	Summary Stats	Missing Rate
Age	subject-level	Median (range)	76 (60, 98)	0%
Smoking status	subject-level	Ever-smoker	40 (17%)	0%
Education	subject-level	High school<br>Some college<br>College graduate	62 (26%)<br>86 (36%)<br>93 (38%)	0%
Metabolic Syndrome	subject-level	Yes	95 (39%)	0%
nteeth	subject-level	Median (range)	22 (1, 28)	0%
CAL	Tooth-level	levels	4	19%
PPD	Tooth-level	levels	4	10%
ABL	Tooth-level	levels	6	25%
Mobil	Tooth-level	levels	4	0.2%

Table S2: Results of intercept η_1 and slope β_1 when ICS=0.4, ICC=0.6, , missing rate was 20%, sample size N was 50, missing mechanism was MAR, $C = 4$.

Parameter	Method	Mean Est	Mean SE	Empirical SE	Rel Bias (%)	Cov Prob (%)	MSE
$\boldsymbol{\eta_1 = -0.4}$							
	Full	-0.38	0.30	0.29	4.58	95.20	0.08
	CCA	-0.10	0.29	0.32	76.03	76.68	0.19
	FCS+CS	-0.35	0.31	0.28	11.53	96.59	0.08
	FCS	-0.32	0.32	0.29	19.78	94.46	0.09
	JM+CS	-0.32	0.34	0.30	19.62	95.61	0.10
	JM	-0.29	0.34	0.31	26.71	93.45	0.11
$\boldsymbol{\beta_1 = -0.2}$							
	Full	-0.22	0.25	0.29	-8.30	90.70	0.08
	CCA	-0.10	0.28	0.34	50.82	87.34	0.13
	FCS+CS	-0.20	0.29	0.28	-1.95	95.58	0.08
	FCS	-0.17	0.30	0.29	17.16	95.87	0.08
	JM+CS	-0.15	0.34	0.33	24.51	95.61	0.11
	JM	-0.12	0.34	0.31	38.12	96.12	0.10

Table S3: Results of intercept η_1 and slope β_1 when ICS=0.1, ICC=0.3, , missing rate was 20%, sample size N was 50, missing mechanism was MCAR, $C = 4$.

Parameter	Method	Mean Est	Mean SE	Empirical SE	Rel Bias (%)	Cov Prob (%)	MSE
$\boldsymbol{\eta_1 = -0.4}$							
	Full	-0.40	0.20	0.20	-0.97	95.50	0.04
	CCA	-0.40	0.21	0.21	-1.03	94.90	0.05
	FCS+CS	-0.40	0.21	0.20	-0.05	95.30	0.04
	FCS	-0.40	0.21	0.20	0.48	95.50	0.04
	JM+CS	-0.40	0.21	0.21	-0.74	95.80	0.04
	JM	-0.40	0.21	0.21	-0.91	95.70	0.04
$\boldsymbol{\beta_1 = -0.2}$							
	Full	-0.21	0.19	0.20	-4.48	92.40	0.04
	CCA	-0.21	0.20	0.22	-6.43	92.30	0.05
	FCS+CS	-0.21	0.19	0.20	-6.42	95.10	0.04
	FCS	-0.21	0.19	0.20	-5.89	94.20	0.04
	JM+CS	-0.21	0.20	0.21	-5.19	94.00	0.04
	JM	-0.21	0.20	0.21	-3.96	94.20	0.04

3 Supplementary Figures

Figure S1: Relationship between mean clinical attachment loss (CAL) score (0: < 2mm, 1: 2-2.9mm,2: 3-4.9mm, 3: $\geq$ 5mm) and number of teeth per participant from Department of Veterans Affairs Longitudinal Dental Study (N=241).

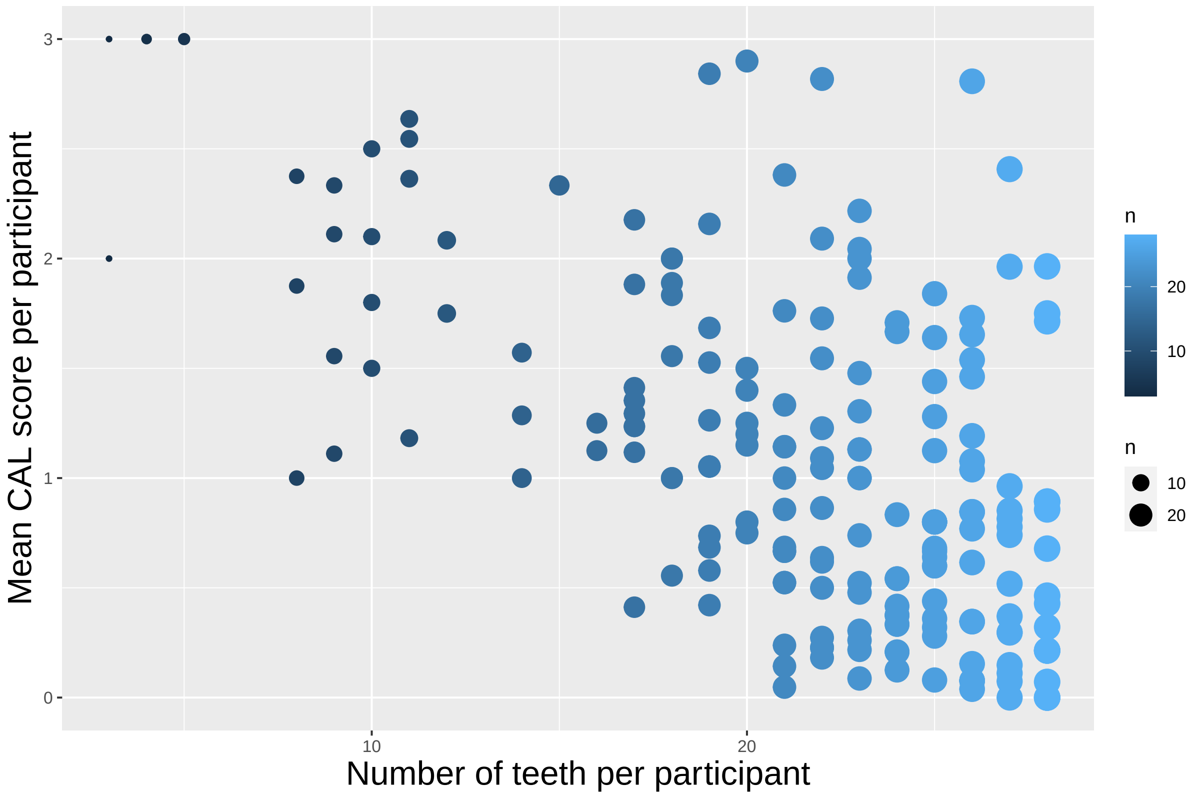

Figure S2: Mean relative bias of each imputation method and each parameter under different simulation scenarios. The missing data mechanism was MAR and $C = 4$. The missing rate was 50%. Each column in Figure 1 represents one combination of parameters of interest, degrees of ICS, and ICC, with two different sample sizes. The black line is the reference line at 0; the grey line represents the results using the full data; the green line represents the results using complete case analysis; the blue line represents the results using FCS+CS; the red line represents the results using FCS; the purple line represents the results using JM+CS; the orange line represents the results using JM.

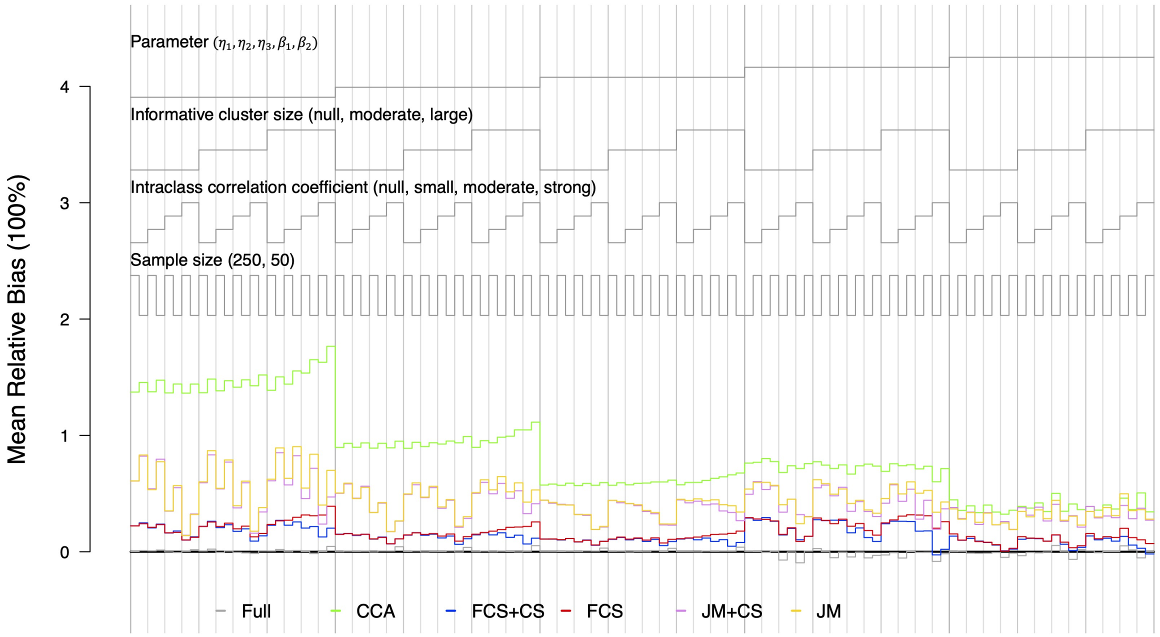

Figure S3: Mean relative bias of each imputation method and each parameter under different simulation scenarios. The missing data mechanism was MAR and $C = 3$. The missing rate was 20% and the sample size was 50. Each column represents one combination of parameters of interest and degrees of ICS, with four different values of ICC. The black line is the reference line at 0; the grey line represents the results using the full data; the green line represents the results using complete case analysis; the blue line represents the results using FCS+CS; the red line represents the results using FCS; the purple line represents the results using JM+CS; the orange line represents the results using JM.

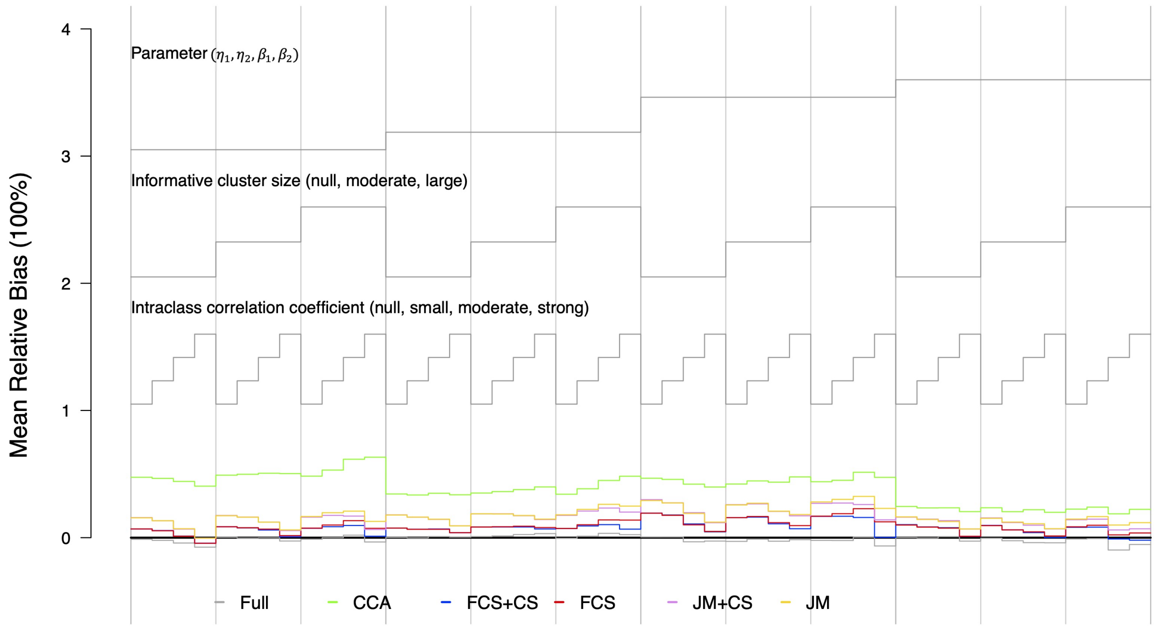

Figure S4: Mean relative bias of each imputation method and each parameter under different simulation scenarios. The missing data mechanism was MAR and $C = 4$. The missing rate was 20% and the sample size was 50. The ancillary variables were removed in the imputation model. Each column represents one combination of parameters of interest and degrees of ICS, with four different values of ICC. The black line is the reference line at 0; the grey line represents the results using the full data; the green line represents the results using complete case analysis; the blue line represents the results using FCS+CS; the red line represents the results using FCS; the purple line represents the results using JM+CS; the orange line represents the results using JM.

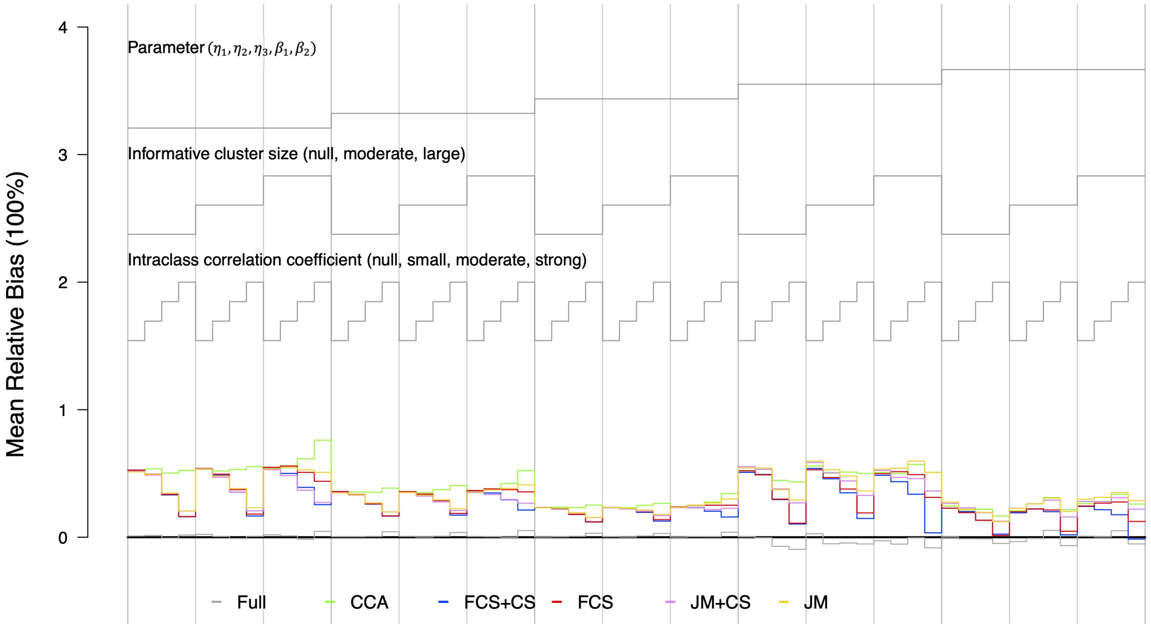

Figure S5: Mean relative bias of each imputation method and each parameter under different simulation scenarios. The missing data mechanism was MCAR and $C = 4$. The missing rate was 20% and the sample size was 50. Each column represents one combination of parameters of interest and degrees of ICS, with four different values of ICC. The black line is the reference line at 0; the grey line represents the results using the full data; the green line represents the results using complete case analysis; the blue line represents the results using FCS+CS; the red line represents the results using FCS; the purple line represents the results using JM+CS; the orange line represents the results using JM.

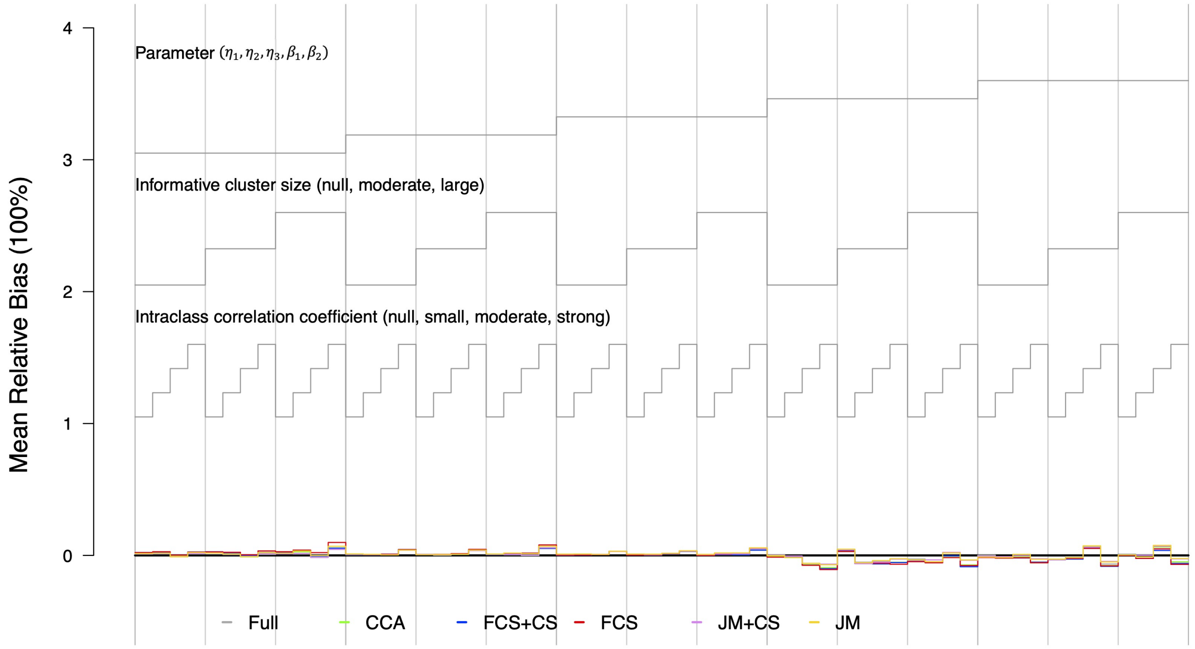

Supplement: Supplementary file 1 — Additional file 1. [file 12874_2023_1909_MOESM1_ESM.pdf]
